# Interception of visible and occluded ballistic trajectories: differential contributions by areas MT/V5, V6, V6A and the vestibular premotor cortex evaluated by TMS

**DOI:** 10.3389/fnint.2026.1907398

**Published:** 2026-09-01

**Authors:** Sergio Delle Monache, Gianluca Paolocci, Michela Gamberini, Francesco Scalici, Iole Indovina, Patrizia Fattori, Claudio Galletti, Francesco Lacquaniti, Gianfranco Bosco

**Affiliations:** 1 Department of Systems Medicine and Centre for Space BioMedicine, University of Rome Tor Vergata, Rome, Italy; 2 Laboratory of Neuromotor Physiology, LIFE Institute for Research and Health Care Santa Lucia IRCCS, Rome, Italy; 3 Department of Medical, Surgical and Technological Sciences, University of Catania, Catania, Italy; 4 Department of Biomedical and Neuromotor Sciences, University of Bologna, Bologna, Italy; 5 Interdepartmental Center for Industrial Research-Aerospace, University of Bologna, Forlì, Italy

**Keywords:** internal models, manual interception, prediction, TMS, visual extrapolation

## Abstract

**Introduction:**

Several cortical regions have been implicated in the control of interceptive actions, such as area MT/V5, posterior parietal areas, a vestibular area in the temporo-parietal junction (TPJ) involved in an internal representation of gravity, and premotor areas. Here, we assessed the potential contributions of visual areas V6 and V6A and of another premotor region in the inferior frontal gyrus associated to the vestibular network (IFG), which could potentially contribute based on their connectivity and known functional properties, and compared them with those of MT/V5.

**Methods:**

We applied triple-pulse transcranial magnetic stimulation (tpTMS) during interception of ballistic trajectories, either congruent with natural gravity (1g) or perturbed with constant-velocity profiles (0g). Trajectories were either fully visible or occluded prior to landing. tpTMS was applied on each cortical site right after the trajectory perturbations or occlusions.

**Results:**

tpTMS of MT/V5 produced robust changes in the interceptive performance with both visible and occluded trajectories, by modulating central processing delays with continuously available visual information, while contributing to visual extrapolation with occluded motion. V6 stimulation produced significant effects only with visible trajectories by modulating the central processing delays downstream of MT/V5. IFG showed distinct contributions to the spatial and temporal aspects of the interceptive action with respect to the integration of predictive signals based on visual information and on internalized gravity information. V6A stimulation was seemingly ineffective.

**Conclusion:**

Altogether, the functional dissociations emerging among these cortical areas support a complex, parallel distributed organization of the cortical network involved in the interception of projectile motion.

## Introduction

The ability to interact with moving objects is fundamental to common behaviors, from catching a ball on the fly to avoiding collisions during navigation. Such actions require that visual information about the object motion is transformed in precise and timely motor commands (Bootsma and van Wieringen, 1990; Regan, 1997; Tresilian, 2004, 2005; Dessing et al., 2005; Zago et al., 2009; Marinovic et al., 2009a,b). Because of the strict temporal constraints of interceptive actions, it is generally believed that incoming sensory information is combined with predictive spatial and temporal estimates of the interception event, necessary to overcome delays inherent to the sensorimotor transformations (Brenner et al., 1998; Brenner and Smeets, 2018; Tresilian, 1990, 1995, 1999; McLeod, 1987; Zago et al., 2008, 2009; Soechting and Flanders, 2008; de Lussanet et al., 2001; Katsumata and Russell, 2012; DeLucia et al., 2003; de la Malla et al., 2014; de la Malla and López-Moliner, 2015; López-Moliner et al., 2007; Gómez and López-Moliner, 2013; Márquez et al., 2024; Treviño et al., 2026). Moreover, integration between sensory and predictive information may occur in a context-dependent manner (Márquez and Treviño, 2025). When visual motion information about the object trajectory is incomplete, degraded, or ambiguous, however, the reliance on predictive mechanisms is increased to warrant successful interception (Bosco et al., 2012; Bosco G. et al., 2015; Delle Monache et al., 2015; La Scaleia et al., 2014, 2015; Russo et al., 2017; Reid and Dessing, 2018; Zago and Lacquaniti, 2005; Villavicencio et al., 2024). The information contributing to the predictive estimates of the interceptive event is multifaceted, drawing on short-term memory of recent sensory inputs, categorical cues, as well as internal models of object dynamics derived from prior experience (Soechting et al., 2009; Dessing et al., 2009; DeLucia and Liddell, 1998; DeLucia, 2004; Makin et al., 2008, 2009; de Rugy et al., 2012; Lacquaniti et al., 2015).

From a neurophysiological standpoint, several brain areas may contribute to the predictive processes underlying the control of interceptive actions. For example, the middle temporal visual area (MT/V5), which plays an essential role in the processing of visual motion information (Newsome et al., 1985; Mikami et al., 1986; Britten et al., 1993; Liu and Newsome, 2003; Tootell et al., 1995; Seiffert et al., 2003; Zimmermann et al., 2011; Maus et al., 2013b), contributes not only to visual motion perception (Newsome and Paré, 1988; Salzman et al., 1990, 1992; Nichols and Newsome, 2002; Britten et al., 1992; Laycock et al., 2007; Campana et al., 2002, 2006; Sack et al., 2006), but also to the predictive control of interceptive actions, since TMS studies have reported causal relationships with the timing of the interceptive responses (Bosco G. et al., 2008; Dessing et al., 2013; Delle Monache et al., 2017).

Downstream of MT/V5, area V6, which represents wide-field visual motion in retinotopic coordinates (Galletti et al., 1999a; Pitzalis et al., 2010, 2013a; Cardin et al., 2012), and its neighbor visuomotor area V6A, which integrates visual, somatosensory and motor signals (Galletti et al., 1999b,^2003^, ^2022^; Pitzalis et al., 2013b; Bosco G. et al., 2015), have been associated with the online guidance of goal-directed actions, such as pointing and grasping movements (Galletti et al., 1999b; Santandrea et al., 2018; De Vitis et al., 2025; Fattori et al., 2005, 2009, 2010, 2012, 2017; Breveglieri et al., 2014; Hadjidimitrakis et al., 2014; Bosco A. et al., 2015). Remarkably, the functional connectivity of area V6A has been shown to increase during tasks requiring temporal and spatial predictions of occluded parabolic trajectories, suggesting a potential role also in the control of interceptive actions (Zbären et al., 2024). Except for this study, however, the specific contributions of areas V6 and V6A to the control of interceptive actions remain largely unexplored.

Predictive signals for the control of manual interceptive actions may arise also from areas in the posterior parietal cortex. Persistent activity during visual occlusion of moving targets in areas of the intraparietal sulcus (IPS), such as the non-human primate area LIP, has been associated to visual extrapolation processes (Assad and Maunsell, 1995; Eskandar and Assad, 1999; Olson et al., 2004; Maimon and Assad, 2006; Shuwairi et al., 2007). Moreover, it has been shown that IPS activity can causally influence the timing of interceptive responses to occluded ballistic trajectories, supporting its role in the control of interceptive actions (Delle Monache et al., 2017). Furthermore, neuronal activities in area 7a can explicitly reflect predictive information about the impending interceptive action (Merchant et al., 2004a; Li et al., 2022).

With regard to the internal models of the object dynamics potentially involved in the control of interceptive actions, it has been suggested that, through lifetime experience, the brain develops an internal representation of the effects of gravity on the objects’ motion, engaged by both perceptual and motor processes, including interceptive actions (Lacquaniti and Maioli, 1989; McIntyre et al., 2001; Senot et al., 2005, 2012; Lacquaniti et al., 2013, 2014, 2015; Le Séac’h et al., 2010; Zago et al., 2008, 2009; Moscatelli and Lacquaniti, 2011; Gravano et al., 2017; Jörges and López-Moliner, 2017; De Sá Teixeira et al., 2019; Delle Monache et al., 2019, 2021, 2023, 2025, 2026; Aguado and López-Moliner, 2021, 2025; Velado et al., 2024).

Consistent neuroimaging evidence has indicated that internalized gravity information is elaborated and stored within the vestibular network, an ensemble of interconnected multimodal cortical and subcortical regions, comprising mainly perisylvian areas, such as the insula, the retroinsula, the parietal operculum, the temporo-parietal junction, involved in the processing of vestibular signals (Guldin and Grüsser, 1998; Brandt and Dieterich, 1999; Indovina et al., 2005; Lopez and Blanke, 2011, Lopez et al., 2012; Goldberg et al., 2012, Wijesinghe et al., 2015). Vestibular network regions, in fact, can show preferential activations in response to visual motion congruent with the effects of Earth gravity (Indovina et al., 2005; Indovina et al., 2013a,b; Indovina et al., 2015; Miller et al., 2008; Maffei et al., 2010; Ferri et al., 2016). Furthermore, TMS applied to a core region of the vestibular network in the temporo-parietal junction (TPJ), located in the supramarginal gyrus at the apex of the sylvian sulcus, has revealed causal links with the timing of the interceptive responses to object motion congruent with gravity (Bosco G. et al., 2008; Delle Monache et al., 2017).

The vestibular network extends beyond parieto-temporal areas around the Sylvian sulcus, by including motor and premotor areas, such as the frontal eye fields (FEF), the supplementary eye fields, the ventral premotor cortex in the inferior frontal gyrus, and the anterior cingulate portion of the supplementary motor area (SMA), thereby creating a direct link between vestibular and gravity-related information and motor behaviors (Brandt and Dieterich, 1999; Dieterich and Brandt, 2024; Lobel et al., 1998; Indovina et al., 2020; Raiser et al., 2020; Delle Monache et al., 2021, Graf, 2025). Notably, a vestibular premotor region in the inferior frontal gyrus, including Broadmann area 44 (Amunts et al., 2020), has been shown to be activated preferentially by visual motion congruent with gravity, suggesting a contribution in the control of the interceptive action as demonstrated for other areas of the vestibular network such as the TPJ (Indovina et al., 2005; Delle Monache et al., 2017). However, specific evidence concerning the involvement of this premotor site in the control of interceptive action is lacking. Most primate neurophysiology and human neuroimaging studies have focused on the dorsal premotor cortex and on the SMA, showing that activity in these areas can be related to predictive timing (Merchant et al., 2004b,^2009^; Merchant and Averbeck, 2017; Merchant and Georgopoulos, 2006; Mita et al., 2009; Tombini et al., 2009; Field and Wann, 2005; de Azevedo Neto and Amaro Júnior, 2018). Moreover, TMS applied to a dorsal premotor cortex site has been shown to alter a working memory trace accounting for the serial dependence of interceptive responses to successive trials with different target speeds (de Azevedo Neto and Bartels, 2021).

In sum, although a relevant body of neurophysiological and neuroimaging work has identified several cortical regions as key players in the control of interceptive actions, experimental evidence for other visual and visuomotor areas such as V6 and V6A, as well as for the vestibular premotor region in the inferior frontal gyrus (IFG), is lacking, despite their connectivity pattern and overall functional properties suggest they may contribute as well.

To address this gap, we applied triple-pulse transcranial magnetic stimulation (tpTMS) over MT/V5, V6, V6A, and IFG while participants intercepted visual targets moving along ballistic trajectories. The present study represents, to our knowledge, the first attempt to assess causal relationships between the activities of V6, V6A, and IFG and the control of interceptive actions. Instead, for area MT/V5 the aim was extending earlier findings (Bosco G. et al., 2008; Delle Monache et al., 2017; Dessing et al., 2013), as well as comparing directly the effects of its stimulation with those of the visual motion area V6, which resembles area MT/V5 for many functional aspects (Pitzalis et al., 2010, 2013a,c).

To determine whether the participants’ interceptive responses could reflect prior knowledge of gravity’s effects, we manipulated the target motion to either follow the natural gravitational law of motion or to shift abruptly to a constant-velocity (0g) profile. Moreover, to enforce predictive mechanisms, in separate experimental sessions, the moving target was either continuously visible or occluded for variable intervals before landing. By applying tpTMS shortly after either the trajectory perturbation or occlusion, we sought to assess the causal role of each cortical site in shaping the spatial and temporal aspects of the interceptive responses both when visual feedback of the target motion was continuously available and under the visual occlusion condition requiring extrapolation of the target motion.

We found novel evidence for functional dissociations among these areas, with distinct contributions of areas MT/V5, V6, V6A, and IFG to the visuomotor transformation and visual extrapolation processes underlying temporal and spatial control of the interceptive action, supporting the parallel distributed organization scheme proposed by Delle Monache et al. (2017) for the cortical network potentially involved in interceptive control.

## Materials and methods

Thirteen subjects (7 women, 6 men, mean age ± SD: 30.8 ± 4.9) participated in two experiments with identical experimental conditions, but during which different cortical areas of the left hemisphere were submitted to Transcranial Magnetic Stimulation (TMS): visual areas MT/V5 and V6 in the first experiment; visuomotor area V6A and a premotor cortical site in the inferior frontal gyrus (IFG) belonging to the vestibular network in the second experiment. For safety reasons, at least 6 months elapsed between the two experiments (Rossini et al., 2015). All participants had normal or corrected-to-normal vision and were classified as right-handed according to the abbreviated version of the Edinburgh Handedness Inventory (mean ± SD laterality quotient: 0.76 ± 0.14) (Oldfield, 1971). None reported any neurological disorders, and all met the safety criteria for TMS studies (Rossini et al., 2015). Participants provided written informed consent prior to participation and received a monetary reimbursement of 50 Euros per experiment. The experimental procedures complied with the Declaration of Helsinki and were approved by the Ethics Committee of the University of Rome “Tor Vergata” (R.S. 27.19).

### Visual scene and ball trajectories

The experimental setup, including the visual scenes and some of the ball trajectories, was adapted from Delle Monache et al. (2017). Participants sat 60 cm from a 22-inch LCD monitor (ViewSonic VX2268WM) with their heads stabilized by a chin rest. Visual scenes were created using the software “Presentation” (version 14.9; Neurobehavioral Systems) and displayed at a refresh rate of 100 Hz with a spatial resolution of 1,680 × 1,050 pixels, spanning 42.86° × 26.79° of visual angle.

**FIGURE 1:**
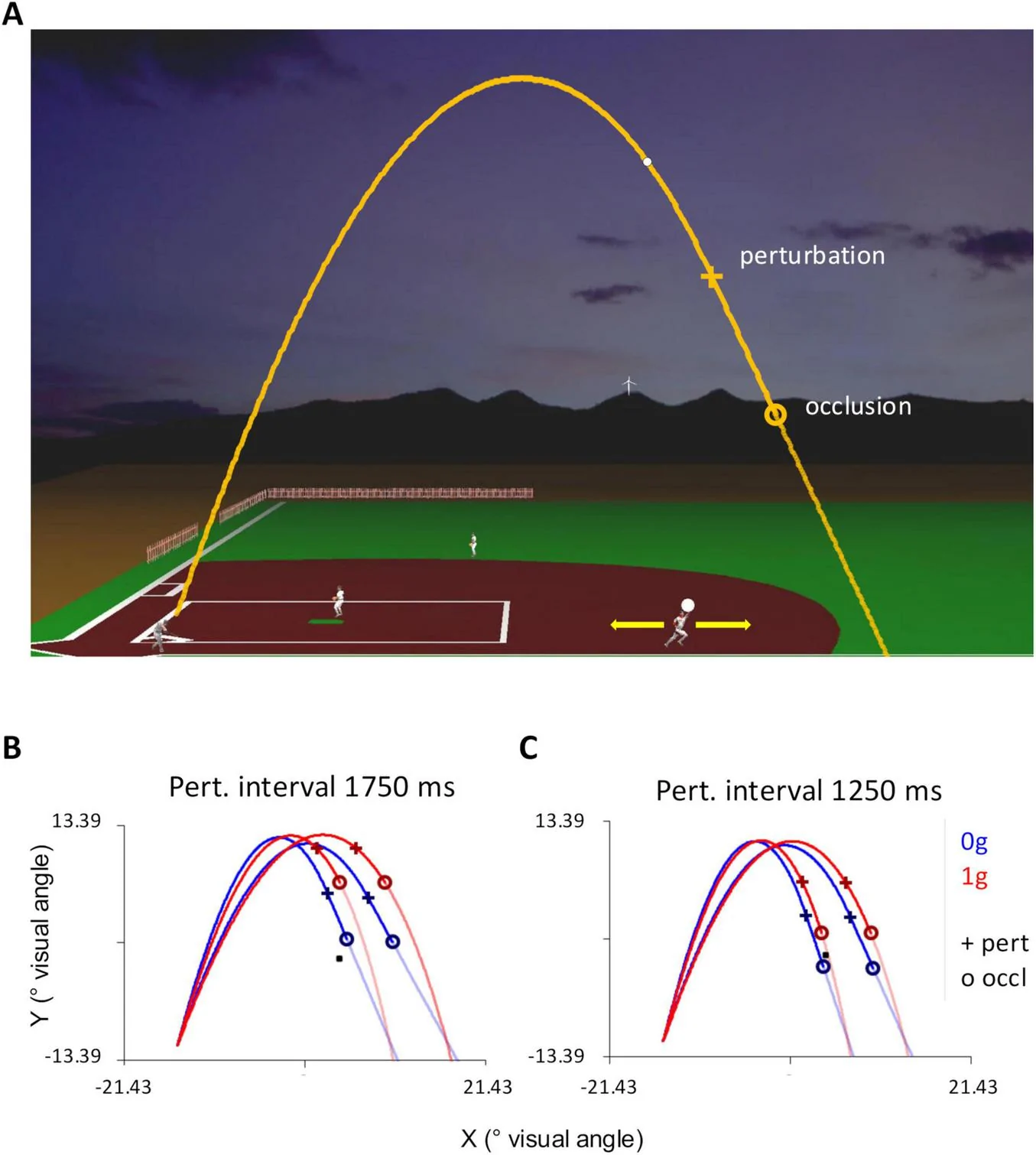
Visual scene and ball trajectories. **(A)** The visual scene simulated the fly-ball play of the baseball game. The orange trace represents one of the perturbed 0g trajectories (the ball is indicated by the white circle), with the perturbation event indicated by a cross. Participants intercepted the ball by displacing the outfielder (see yellow arrows) with a computer mouse and by pressing the left mouse button at the time of interception. The semitransparent circle on the outfielder’s hand delimited the area within which interception was considered successful. During the *Visible Session*, trajectories were entirely visible until landing. During the *Occluded Session*, the ball disappeared 500 ms after the trajectory perturbation event (the circle denotes the occlusion onset and the dotted segment describes the invisible portion of the trajectory). **(B)** 0g trajectories (blue traces) perturbed 1,750 ms before landing and unperturbed 1g trajectories (red traces) matched for the landing location. Crosses and open circles indicate the trajectory perturbation and occlusion onsets, respectively. Note that for 1g trajectories equivalent temporal markers of the perturbation were defined to apply the TMS pulses and the visual occlusions at corresponding times as the perturbed 0g trajectories. The light-colored trace denotes the invisible portion of the trajectory after the onset of the occlusion. The black dot indicates the ocular fixation point. **(C)** Same as **(B)** for 0g trajectories perturbed 1,250 ms before landing and corresponding unperturbed 1g trajectories.

Scenes depicted a baseball game in which a ball (diameter: 7 pixels, 0.18° visual angle), hit by a batter on the left side, followed a quasi-parabolic trajectory and landed at the bottom right corner of the display (Figure 1A). The ascending portion of the trajectory was modeled by considering the effects of gravity and air drag, whereas the descending portion could either be unaltered maintaining the gravitational acceleration (unperturbed 1g motion) or be perturbed by assuming abruptly a constant velocity kinematic profile, simulating a zero-gravity condition (0g motion). In different trials, the perturbation could occur at two distinct time-points along the trajectory: either 1750 ms (Figure 1B) or 1250 ms (Figure 1C) prior to the ball landing. For the unperturbed 1g trials, equivalent temporal markers were defined to apply the TMS pulses and the visual occlusions at corresponding times as the perturbed 0g trajectories (see below).

Four perturbed 0g perturbed trajectories (blue traces in Figures 1B,C) were created by adopting two different combinations of initial velocities (V_0_) and launch angles for each perturbation interval: for trajectories perturbed 1,750 ms before landing, we paired V_0_ values of 26.1 and 26.4 °/s with launch angles of 74.4° and 69.8°, respectively; for trajectories perturbed 1,250 ms before landing, V_0_ values of 25.3 and 25.8 °/s were paired with launch angles of 75.4° and 70.6°, respectively. This trajectory design was instrumental to span the ball landing locations across the right side of the visual scene. Similarly, unperturbed 1g trajectories (red traces in Figures 1B,C) were launched with four possible combinations of V_0_ and launch angle values (25.5, 26.3, 26.5, or 27.5°/s paired with 74.5°, 69.7°, 72.8°, or 68.3°), thus allowing to match the spatial distribution of the landing locations of the 0g trajectories and avoid spatial clustering of 0g and 1g trajectories. The resulting eight trajectories are shown separately for the two perturbation intervals of 1,750 and 1,250 ms in Figures 1B,C, where trajectory perturbation and occlusion onsets are denoted, respectively, by crosses and circles. The experimental manipulations of V_0_ and perturbation interval were introduced merely to increase the number of experimental conditions as well as the spatial span of the ball trajectories and were not analyzed further, since the study focused on the effects of tpTMS applied to the four cortical areas on the interception of trajectories either congruent or not with gravity. This choice was also supported by previous evidence, obtained with a similar experimental setup, that the perturbation interval was not a significant factor in explaining the changes in the interceptive responses following tpTMS (Delle Monache et al., 2017).

Each trial began with the image of a running outfielder centered at the bottom of the scene. Although both the ball and the outfielder moved in the frontal plane, depth and scale information were provided through perspective and background cues. Each experiment consisted of two sessions conducted on separate days: a *Visible Session*, in which the ball trajectory was fully visible, and an *Occluded Session*, in which the ball disappeared 500 ms after the perturbation temporal marker.

### Interception task

Participants used a gaming mouse (Razer copperhead, Razer) to move horizontally the outfielder toward the expected ball landing location and intercept the falling ball by pressing the left mouse button when the outfielder’s hand intersected the falling ball. A white semi-transparent circle (0.56° visual angle) indicated the valid interception zone. Participants maintained gaze on a designated fixation point—a white windmill in the upper right quadrant of the scene (0.4° visual angle)—positioned slightly above the interception height. The location of the windmill was such that the trajectory perturbations occurred between 4.9 and 12.8 degrees of visual angle from the fixation point, while the landing locations were comprised between 9.8 and 15.9 degrees of visual angle from the fixation point.

Mouse cursor position was sampled at 200 Hz via USB, while button presses were recorded at 1 kHz (CED Power 1401 system running Spike2 software). Eye position was monitored at 500 Hz using an EyeLink 1000 tracker (SR Research). Eye tracker calibration was performed at the start of each block, with drift corrections every 18 trials.

Before the main experiment, participants familiarized with the task by performing one training block in a simplified visual scene with a dark background and a red rectangular launcher at the top (0.61° visual angle). The launcher fired a spherical object (0.18° visual angle) downward along one of nine rectilinear trajectories determined by launcher position, angle, initial velocity, and acceleration. The training block comprised 45 trials (five repetitions per trajectory in pseudo-random order).

### Transcranial magnetic stimulation

**FIGURE 2:**
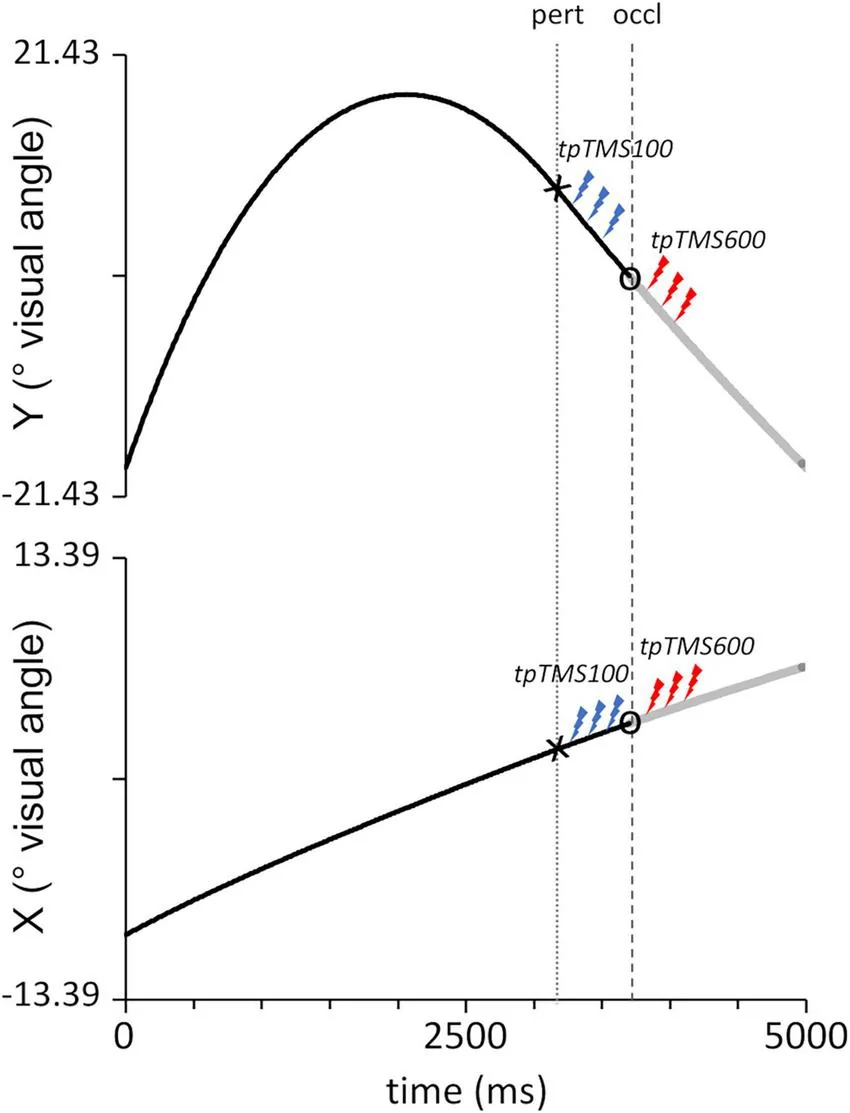
tpTMS paradigm. Bottom and top panels represent the time course of the X and Y coordinates of a perturbed 0g trajectory, respectively. Vertical dotted and dashed gray lines signify the occurrence of the 0g perturbation (pert) and of the ball disappearance (occl), respectively. Three TMS pulses, indicated by the thunderbolts, were applied at a frequency of 10 Hz, either 100 ms (blue thunderbolts; *tpTMS100*) or 600 ms (red thunderbolts; *tpTMS600*) after the trajectory perturbation. Note that during the *Occluded Session*, *tpTMS600* occurred 100 ms after the ball disappearance.

Cortical activity was transiently disrupted by using triple-pulse TMS (tpTMS; three pulses at 10 Hz; see Sack et al., 2005; Striemer et al., 2011). Stimulation was delivered through a figure-of-eight coil (70 mm; Magstim SuperRapid2, United Kingdom) at 70% of maximum output (Delle Monache et al., 2017). Because the effects of a single TMS pulse may last ∼50–100 ms (Pascual-Leone et al., 2000; Siebner and Rothwell, 2003; Striemer et al., 2011; Grotheer et al., 2016; Wang et al., 2016), tpTMS likely modulated cortical excitability for approximately 300 ms (Sack et al., 2005; see also Delle Monache et al., 2017). tpTMS was applied on two-thirds of trials, either 100 ms (*tpTMS100*) or 600 ms (*tpTMS600*) after the perturbation onset, or at corresponding time markers for unperturbed 1g trajectories (Figure 2). In the *Occluded Session*, due to the fixed 500 ms delay between perturbation and occlusion, *tpTMS600* occurred 100 ms after ball disappearance. To reduce the potential facilitating effects of TMS auditory artifacts on the motor responses (Pascual-Leone et al., 1992; Terao et al., 1997), participants wore in-ear earphones (Bose, United States) connected to a portable media player, which played continuously a white noise track. Prior to the experiment, we adjusted, with test TMS pulses, the volume of the media player until masking the clicking noise of the magnetic coil discharge, which could be estimated being about 90 dB at 70% of the stimulator output and at a 5 cm distance from the coil (Dhamne et al., 2014).

#### TMS sites and coil positioning

Stimulation sites (MT/V5, V6, V6A, and IFG) on the left hemisphere were defined in MNI stereotactic space based on prior fMRI and TMS studies (Orban et al., 2003; Indovina et al., 2005; Bosco G. et al., 2008; Ciavarro et al., 2013; Delle Monache et al., 2017). The V6 and V6A sites were localized based on the individual cortical anatomy following the morphology of the parieto-occipital sulcus, as described in previous anatomical and functional studies (Pitzalis et al., 2006, 2010, 2013a). A control site, the Vertex, which corresponds to the Cz scalp site in the 10–20 EEG system, was also stimulated to account for somatosensory and auditory artifacts.

Anatomical MRI scans were acquired for each participant with a Siemens Magnetom Allegra 3T scanner (T1-weighted MPRAGE sequence, TR = 2.00 s, TE = 4.38 ms, flip angle = 8°, 1 mm resolution, 176 sagittal slices). MRI volumes were normalized to the MNI T1-256 template using FLIRT (FSL, United Kingdom). Individual MNI coordinates were visualized in MRIcro and mapped back to native space for the neuronavigation procedure.

**FIGURE 3:**
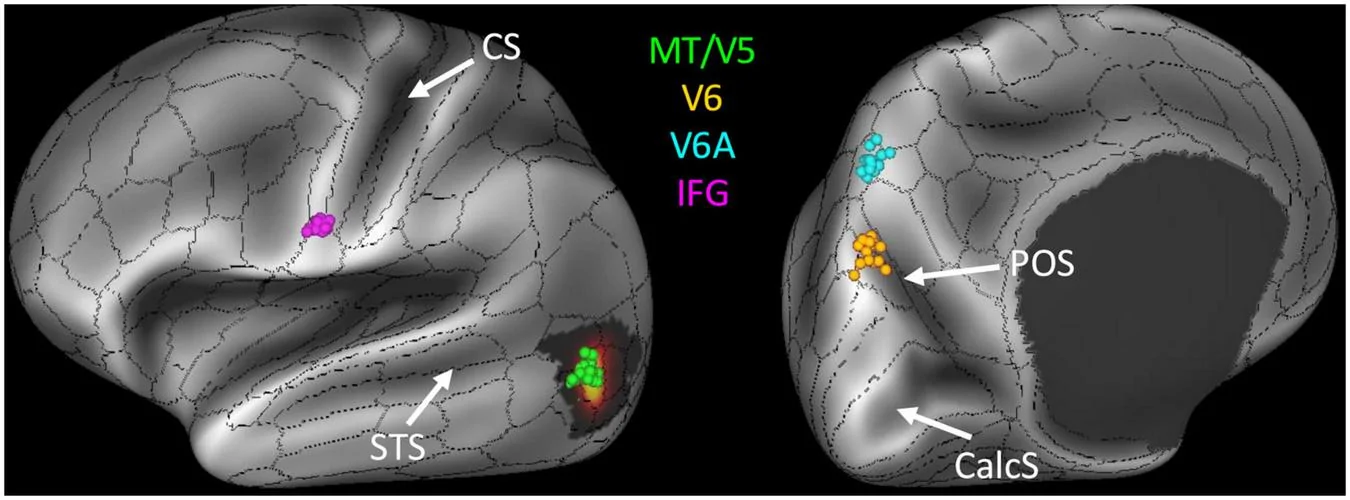
tpTMS site locations. The location of individual target cortical sites stimulated on the left hemisphere of the thirteen participants are represented on a 3D rendering of the HCP1200 brain template (Glasser et al., 2016). tpTMS was applied at the following mean (±SD) MNI coordinates: area MT/V5: - 53.7 ± 1.1, - 75 ± 2.1, 3.3 ± 1.6; area V6: - 7.9 ± 0.6, - 87.1 ± 2.6, 38.3 ± 3.01; area V6A: - 9.3 ± 1.3, - 76.5 ± 1.6, 57 ± 1.7; IFG: - 62.3 ± 1.1, 6.8 ± 1.4, 19.8 ± 0.9. The lateral view (on the left side) shows the stimulation sites for visual motion area MT/V5 (green circles, superimposed on the probabilistic location map of MT/V5 defined by the brain parcellation scheme by Glasser et al., 2016) and IFG (magenta circles). The medial view (on the right side) shows the stimulation sites for visual areas V6 (yellow circles) and V6A (cyan circles). CS, Central Sulcus; STS, Superior Temporal Sulcus; POS, Parieto-occipital Sulcus; CalcS, Calcarine Sulcus.

Coil positioning was neuronavigated (Brainsight, Rogue Research) using each participant’s MRI, with a mechanical arm (Magic Arm, Manfrotto, Italy) holding the coil in place. Coil position was monitored at 20 Hz, and experiments were paused if head movements displaced the coil by more than 3 mm, and the coil was repositioned. MNI coordinates of stimulation sites for the 13 participants are listed in Table 1, and represented, in Figure 3, on a 3D rendering of the left hemisphere of the human brain (Glasser et al., 2016).

**TABLE 1:** MNI coordinates of the target TMS sites for the thirteen participants to the study.

	MT/V5			V6			V6A			IFG		
Subject #	*X*	*Y*	*Z*	*X*	*Y*	*Z*	*X*	*Y*	*Z*	*X*	*Y*	*Z*
1	−54	−73	3	−8	−84	32	−10	−75	57	−60	6	20
2	−52	−76	5	−8	−88	41	−11	−78	54	−62	7	22
3	−54	−74	4	−8	−91	36	−10	−75	61	−63	8	21
4	−54	−77	6	−8	−85	41	−11	−77	58	−63	8	19
5	−55	−73	2	−8	−93	36	−10	−79	58	−63	10	18
6	−55	−76	3	−8	−86	39	−6	−73	58	−63	5	20
7	−53	−76	5	−8	−86	43	−9	−77	55	−62	6	20
8	−52	−70	0	−8	−85	39	−8	−78	58	−63	6	20
9	−54	−78	3	−8	−87	35	−9	−76	56	−63	6	19
10	−53	−76	5	−8	−85	41	−10	−76	56	−62	9	19
11	−55	−75	3	−9	−89	40	−8	−76	56	−60	6	20
12	−53	−77	2	−8	−88	38	−9	−78	57	−63	6	20
13	−55	−76	3	−6	−85	38	−10	−77	57	−63	6	20

### Experimental layout

**FIGURE 4:**
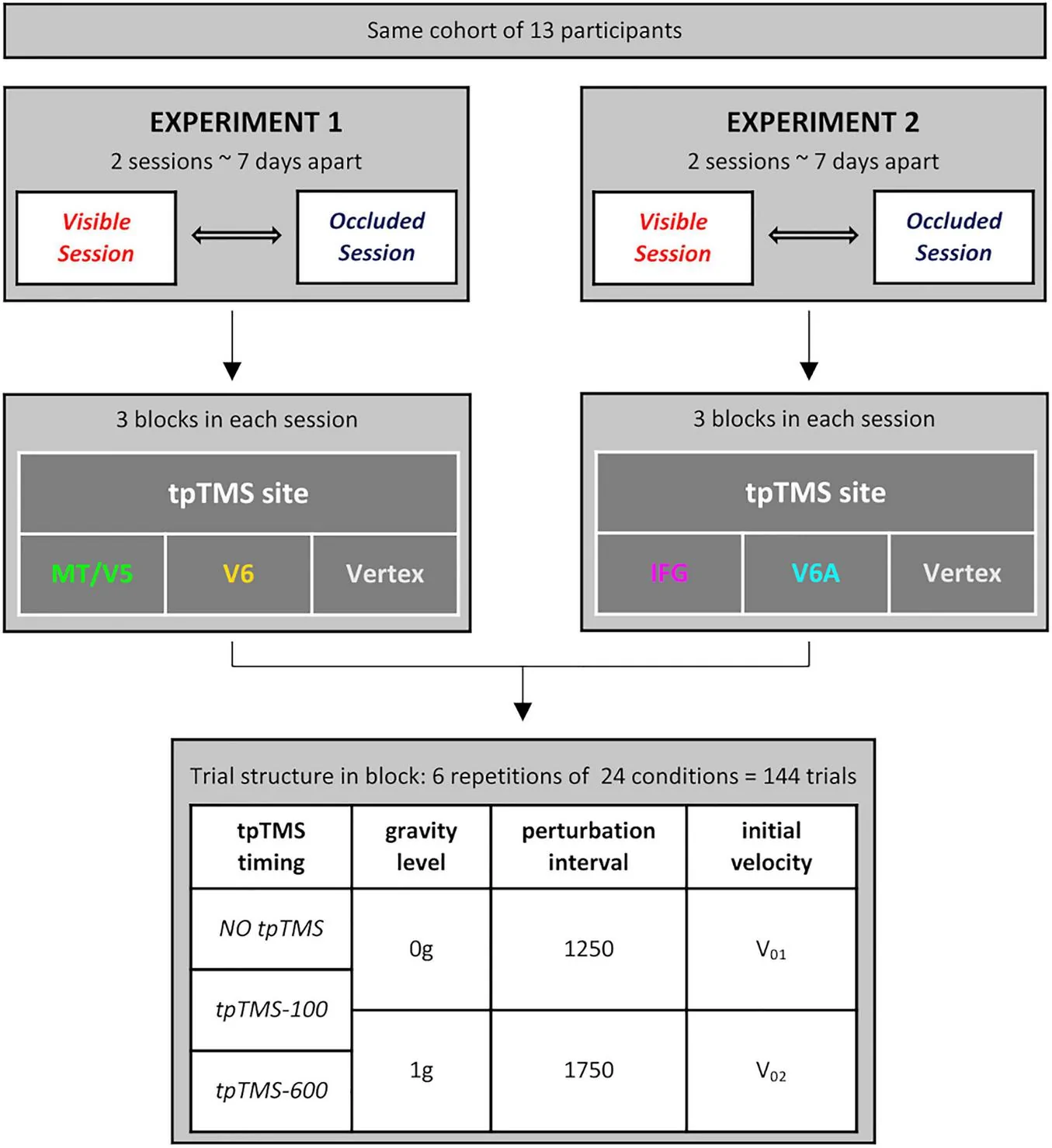
Experimental layout. A cohort of thirteen subjects participated to two experiments held at least 6 months apart. Each experiment comprised two sessions, *Visible Session* and *Occluded Session*, held about one week apart and counterbalanced among participants. Each session comprised three blocks of trials in which different TMS sites were stimulated: MT/V5, V6 and the Vertex in experiment 1; V6A, IFG and the Vertex in experiment 2. Each block contained 144 trials resulting from 6 pseudorandomized repetitions of 24 experimental conditions that manipulated the trajectory Gravity Level, Perturbation Interval and initial velocity as well as the tpTMS timing.

The general experimental layout is illustrated in Figure 4. Each experiment comprised two sessions (*Visible* and *Occluded*) held 7.2 ± 0.7 days apart and performed in counterbalanced order among participants. Each session comprised three blocks, one for each stimulation site, namely MT/V5, V6 and the Vertex for the first experiment and V6A, IFG and the Vertex for the second experiment. For each of the eight trajectories, three TMS conditions were tested:

1. *No-stim*—baseline condition without tpTMS
2. *tpTMS100—*tpTMS applied 100 ms after the trajectory perturbation
3. *tpTMS600—*tpTMS applied 600 ms after the trajectory perturbation

The 24 resulting conditions were repeated six times per block, for a total of 144 trials presented in pseudo-random order.

### Data analysis

All preprocessing was performed using custom MATLAB scripts (Matlab 2020b, Mathworks).

#### Ocular fixation

We monitored participants’ ocular fixation throughout the trials by inspecting the eye-position traces in real-time and by performing ex-post analyses on the eye-tracker data. Ex-post analyses included low-pass filtering (2nd-order Butterworth, zero-lag, cut-off frequency 40 Hz) of the cyclopean eye-position time-series obtained by averaging the left and right eye time-series. Saccades were detected using combined velocity (>30°/s) and acceleration (>2,000°/s^2^) thresholds (Delle Monache et al., 2015). We considered that participants broke fixation when either the ocular drift exceeded 2° of visual angle from the fixation point for more than 200 ms or saccadic movements were detected (Delle Monache et al., 2017). Trials with fixation breaks (449 out of 22,464; 1.9%) were excluded.

#### Interceptive responses

Interception performance was assessed by computing the Temporal Error (TE) and the Position Error (PE) from the button press timing and the mouse cursor position. TE was defined as the difference between the button press time and the moment the ball reached the interception point (positive = late response, negative = early response). Since the outfielder’s movements was confined to the horizontal axis, PE represented the horizontal distance in mm between the outfielder and the ideal interception point (positive = overshoot, negative = undershoot). Mean and standard deviation of TE and PE across repetitions were used as indexes of interception accuracy and precision, respectively.

#### Statistical analyses

Statistical analyses focused on the manipulations of the trajectory acceleration (gravity level) and on the effects of tpTMS applied at the target cortical areas compared to the Vertex. As mentioned earlier, manipulations of the initial ball velocity V_0_ and of the perturbation timing were introduced solely to increase the number of experimental conditions. Thus, data were collapsed across initial velocities and perturbation intervals. For each participant, mean and standard deviation values of TE and PE were computed for 18 reduced conditions (2 gravity levels × 3 TMS conditions × 3 TMS sites) in each session. Outliers exceeding ± 2 standard deviations from the mean were excluded (818 out of 22,464; 3.6%). tpTMS effects were quantified as the change of mean and standard deviation values in each *tpTMS100* and *tpTMS600* condition relative to the corresponding no-stim conditions. These differences will be referred to as ΔTE, ΔsdTE and ΔPE, ΔsdPE for temporal and position errors, respectively.

#### Consistency of the effects of Vertex tpTMS between experiment series

Since the Vertex control site was stimulated during both experiment series, we evaluated the consistency of the effects of the Vertex stimulation with Wilcoxon signed-rank tests, which did not show statistically significant differences between the two experiments (*Visible Session*: ΔTE *p* = 0.127, ΔPE *p* = 0.244; *Occluded Session*: ΔTE *p* = 0.839, ΔPE *p* = 1.0). Based on this finding, we averaged the data of the experimental blocks with Vertex stimulation between the two series for the subsequent statistical models.

#### Generalized linear mixed model (GLMM) analysis

For each experimental session (*Visible*, *Occluded*), mean ΔTE, ΔPE, ΔsdTE, and ΔsdPE pooled from the 13 participants were analyzed using Generalized Linear Mixed Models with “within subject” fixed-effect factors Gravity Level (GL: 0g | 1g), tpTMS Site (V5/MT|V6|IFG|V6A|Vertex), and tpTMS Timing (*tpTMS100*|*tpTMS600*), including also two- and three-way interactions, potentially accounting for response changes evoked by the stimulation of each target cortical site compared to the Vertex, depending on the gravity level and on the timing of tpTMS. Finally, a random effect variable (Subjects) was included to account for variable intercepts across participants. Statistical significance of the GLMM predictors was set at *p* < 0.05. On the significant main effect and interaction effects of tpTMS Site we also performed planned *post-hoc* pairwise tests between each target area and the Vertex control site to determine whether the statistical significance of the predictors involving tpTMS Site was effectively accounted by response changes produced by tpTMS of the target areas compared to the Vertex stimulation. The statistical significance cut-off for the *post-hoc* pairwise comparisons was set to *p* < 0.05, Bonferroni corrected. We performed a-priori sensitivity analyses with the software G*Power to determine the statistical power associated to the *F*-tests of the fixed effects and to the *t*-tests of the *post-hoc* pairwise comparisons. These analyses indicated that the *F*-tests of the fixed effects that included the factor tpTMS site (with degrees of freedom of [4, 240]) had 90% power of detecting as statistically significant, with α cutoff of 0.05, an effect with Cohen’s *d* of 0.49 associated with a noncentrality parameter (δ) of 15.7. All other F-tests having degrees of freedom of [1, 240] had 90% power of detecting as statistically significant, with an α cutoff of 0.05, an effect with Cohen’s *d* of 0.40 and δ of 10.6. Similarly, pairwise tests had 90% power of detecting as statistically significant, with α cutoff of 0.05, an effect size of 0.22 associated with a noncentrality parameter (δ) of 3.25. Statistical analyses were performed by using the statistical software package SPSS v25.0 (IBM, United States).

## Results

The primary objective of the study was assessing the differential contribution of visual areas MT/V5 and V6, visuomotor area V6A, and a premotor site, belonging to the vestibular network, in the Inferior Frontal Gyrus (IFG) to the control of manual interception. For this purpose, we transiently disrupted neuronal activity in each cortical region using tpTMS. GLMMs were employed to test whether tpTMS applied to these cortical areas at two distinct time points during the ball trajectory significantly altered the interceptive performance when visual motion was either congruent or incongruent with natural gravity. Overall, tpTMS applied to MT/V5, IFG and V6 significantly modulated both the timing and spatial accuracy of interception, with effects depending on the congruence of the ball motion with gravity and on whether the ball remained visible or it was occluded. In contrast, stimulation of V6A did not alter significantly the interceptive performance.

### Visible session

**FIGURE 5:**
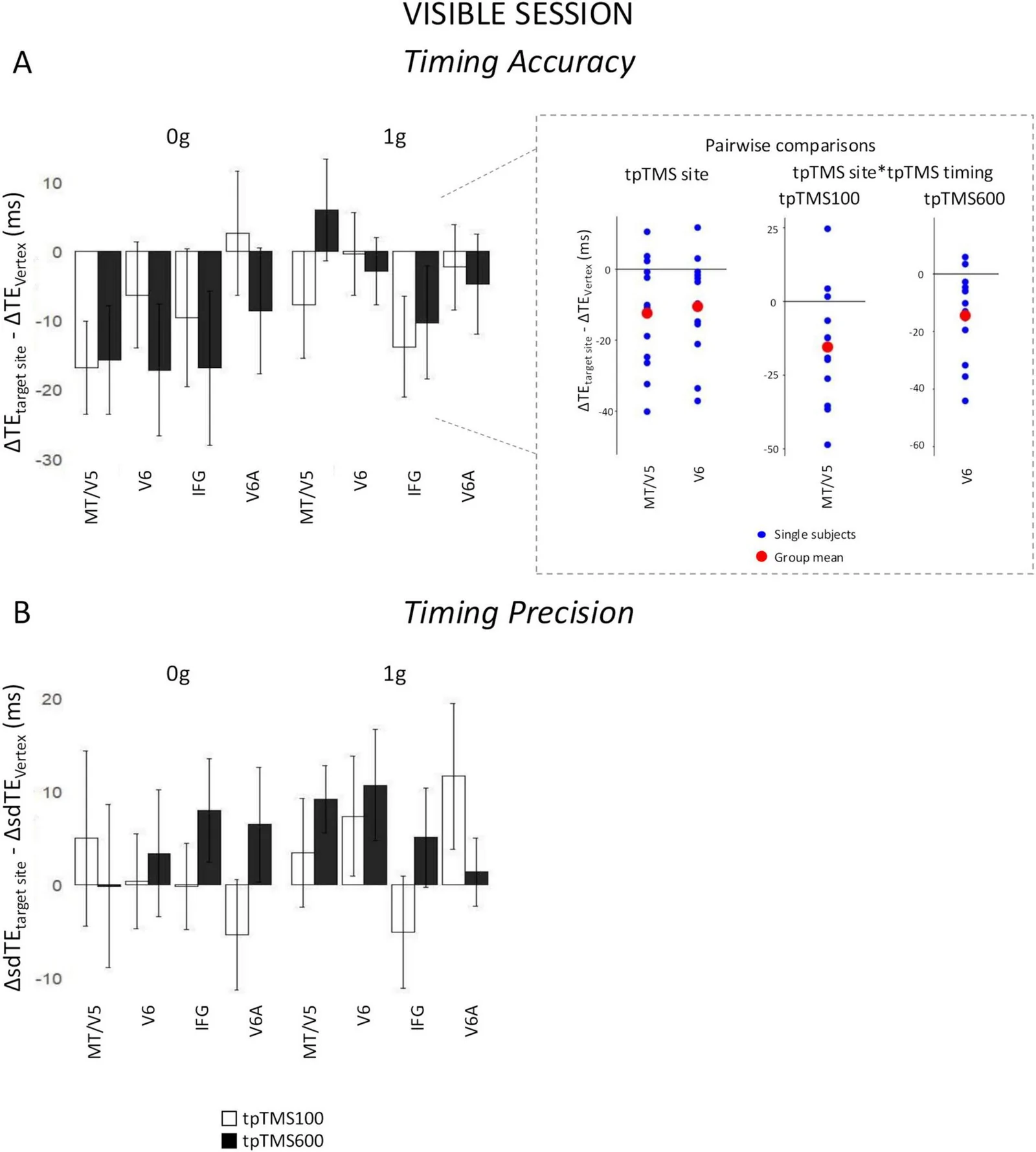
Effects of tpTMS of the four target cortical areas on the interceptive timing observed during the *Visible Session.* The panels in the left column illustrate the effects of *tpTMS100* (empty bars) and *tpTMS600* (black-filled bars) on the interception timing accuracy **(A)** and precision **(B)** following perturbed 0g and unperturbed 1g trajectories. Bars represent mean ΔTEs and ΔsdTEs ( ± SEM) differences between the effects of the stimulation of each target site compared to those of the Vertex computed among the 13 participants. The statistically significant pairwise comparisons between target areas and the Vertex obtained for the main effects of tpTMS site and for the two- and three-way interactions involving tpTMS site are illustrated by the colored graphs in the insets on the right of the main panels. These graphs show with large red symbols the mean ΔTEs and ΔsdTEs differences between the effects of the stimulation of the target areas showing significant effects and those of the Vertex and with smaller blue symbols the mean difference values observed in individual participants for the experimental conditions related to the given effect of interest.

Table 2A reports the outcomes of the GLMM analysis applied to the ΔTE, ΔPE, ΔsdTE, and ΔsdPE datasets drawn from the *Visible Session*. We found that, with fully visible trajectories, tpTMS effects on the interceptive timing accuracy depended strongly on the gravity level, being larger for perturbed 0gthan for unperturbed 1g trials, and on the stimulation site (main effects of GL and tpTMS site in Table 2A and Figure 5A). We examined further the nature of the main effect of tpTMS site by evaluating, with post-hoc pairwise comparisons, whether it was explained by differences between the effects of tpTMS of the target areas compared to the Vertex, given that this latter, on average, shortened slightly the response timing (mean ΔTE = −8.28 ± 3.76 SEM). These *post-hoc* tests, illustrated by the colored inset graphs in Figure 5A, showed that tpTMS of MT/V5 [*t*_(240)_ = −3.04, CI = (−0.98 −23.78), *p* = 0.013] and V6 [*t*_(240)_ = −2.81, CI = (−0.18 −20.81), *p* = 0.015] led to significantly earlier interception responses than Vertex stimulation, thereby implying genuine site-specific effects. The statistically significant two-way interaction tpTMS site*tpTMS timing indicated further that differences among the effects of the TMS sites depended also on the tpTMS timing. In fact, *post-hoc* pairwise tests applied to this interaction effect showed that while MT/V5 stimulation effects were stronger than Vertex with *tpTMS100* [*t*_(240)_ = −3.00, CI = (−1.04 −29.87), *p* = 0.012], significant differences for the effects of V6 [*t*_(240)_ = −3.61, CI = (−3.09 −25.80), *p* < 0.002] emerged only with *tpTMS600* (see the inset colored graphs on the right column of Figure 5A). Finally, larger differences were evident between the effects of tpTMS on 0g and 1g interceptive responses with *tpTMS600* compared to *tpTMS100*, accounting for the statistically significant two-way interaction GL*tpTMS timing (compare the black and the white bars for 0g and 1g trials in Figure 5A, see also Table 2A).

**TABLE 2:** Results of GLMM analyses applied to ΔTE, ΔPE, ΔsdTE, and ΔsdPE datasets.

(A)									
Fixed effects									
		ΔTE		ΔPE		ΔsdTE		ΔsdPE	
Predictor	*df*	*F*	*p*-value	*F*	*p*-value	*F*	*p*-value	*F*	*p*-value
GL	1, 240	11.71	**0.001**	3.14	0.078	0.54	0.462	0.54	0.463
tpTMS site	4, 240	5.93	**< 0.001**	5.79	**< 0.001**	1.21	0.309	1.33	0.259
tpTMS timing	1, 240	0.46	0.500	0.03	0.854	2.55	0.112	0.01	0.934
GL*tpTMS site	4, 240	2.18	0.072	3.90	**0.004**	0.74	0.564	0.70	0.595
GL*tpTMS timing	1, 240	6.48	**0.012**	5.14	**0.024**	0.09	0.765	2.42	0.121
tpTMS site*tpTMS timing	4, 240	4.11	**0.003**	1.54	0.192	1.14	0.338	0.99	0.413
GL*tpTMS site*tpTMS timing	4, 240	1.46	0.214	2.55	**0.040**	2.55	**0.040**	2.40	0.051

**(B)**									
**Fixed effects**									
		**ΔTE**		**ΔPE**		**ΔsdTE**		**ΔsdPE**	
**Predictor**	***df***	***F***	***p*-value**	***F***	***p*-value**	***F***	***p*-value**	***F***	***p*-value**
GL	1, 240	2.04	0.154	0.00	0.978	0.47	0.495	0.99	0.320
tpTMS site	4, 240	3.36	**0.011**	1.58	0.181	0.41	0.800	0.55	0.700
tpTMS timing	1, 240	11.15	**0.001**	4.18	**0.042**	5.34	**0.022**	1.81	0.180
GL*tpTMS site	4, 240	3.11	**0.016**	3.88	**0.004**	0.32	0.864	1.81	0.127
GL*tpTMS timing	1, 240	1.64	0.202	5.01	**0.026**	0.00	0.960	0.01	0.916
tpTMS site*tpTMS timing	4, 240	4.29	**0.002**	1.26	0.288	1.76	0.137	5.96	**< 0.001**
GL*tpTMS site*tpTMS timing	4, 240	2.03	0.090	2.18	0.072	0.91	0.459	1.18	0.319

(A) *Visible session*. (B) *Occluded session*. GL, gravity level. Bold values indicate statistically significant effects (*p* < 0.05).

The precision of the interceptive timing was influenced only by the three-way interaction GL*tpTMS site*tpTMS timing (Figure 5B). However, this three-way interaction was accounted by greater changes occurring following Vertex rather than target sites stimulation and, therefore, it could be considered as resulting from nonspecific effects of the magnetic stimulation.

**FIGURE 6:**
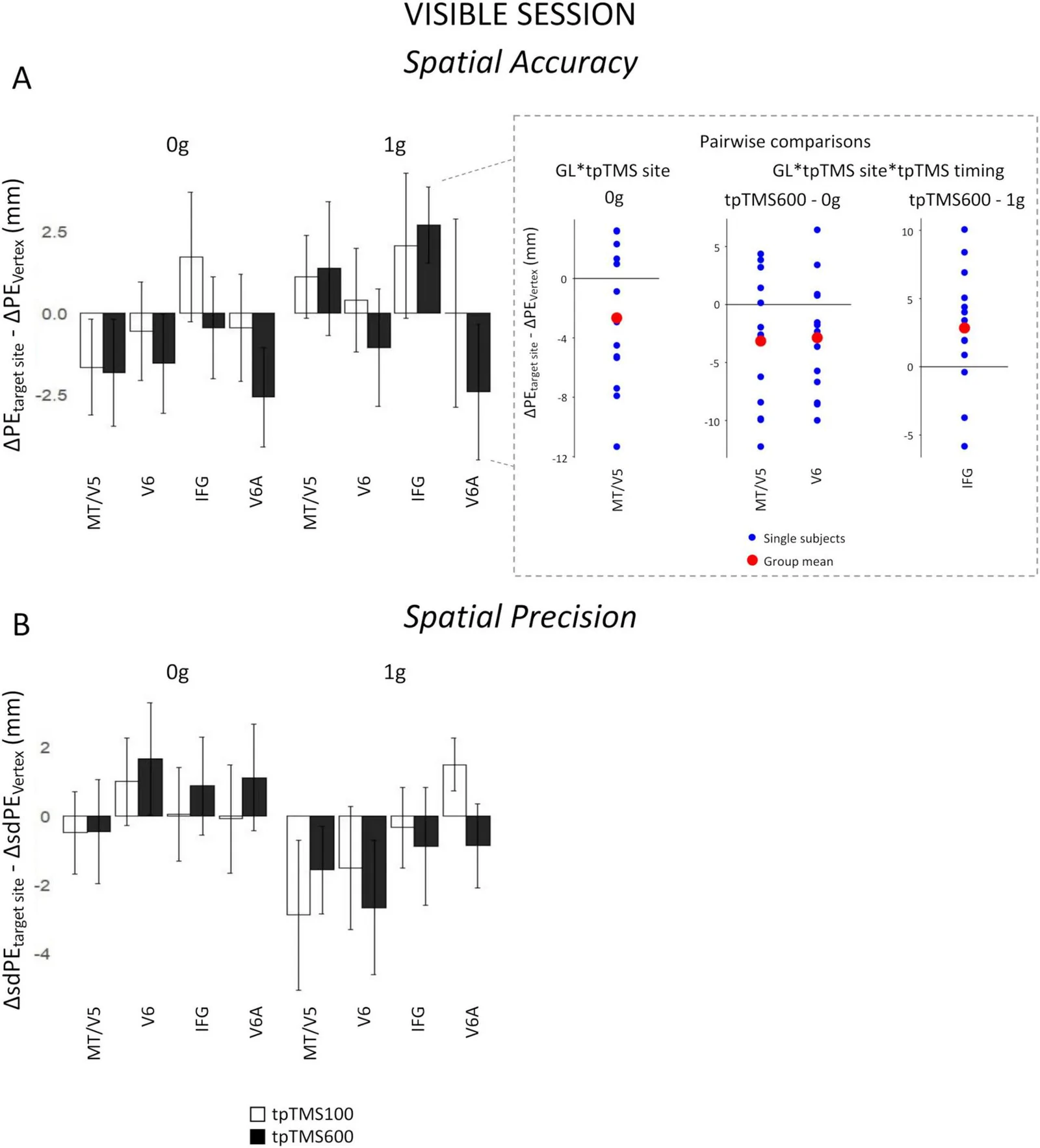
Effects of tpTMS of the four target cortical areas on the interceptive positioning observed during the *Visible Session.* The panels in the left column illustrate the effects of *tpTMS100* (empty bars) and *tpTMS600* (black-filled bars) on the mouse cursor placement accuracy **(A)** and precision **(B)** following perturbed 0g and unperturbed 1g trajectories. Bars represent mean ΔPEs, and ΔsdPEs ( ± SEM) differences between the effects of the stimulation of each target site compared to those of the Vertex computed among the 13 participants. The colored inset graphs illustrate the pairwise comparisons between target areas and the Vertex obtained for the two- and three-way interactions involving tpTMS site that, albeit not surviving Bonferroni corrections, showed *p* < 0.05 by using less restrictive LSD corrections. These graphs show with large red symbols the mean ΔPEs and ΔsdPEs differences between the effects of the stimulation of the target areas showing significant effects and those of the Vertex and with smaller blue symbols the mean difference values observed in individual participants for the experimental conditions related to the given effect of interest.

The main effect of tpTMS site was the largest factor accounting for the accuracy of the mouse cursor placement. To determine whether its statistical significance was explained by differences between the effects of tpTMS on the target areas and the Vertex, we examined the planned pairwise contrasts between target sites and Vertex. These contrasts did not reveal statistically significant differences, suggesting that the main effect of tpTMS site was mostly related to nonspecific effects of the magnetic stimulation (Figure 6A). In addition to the main effect, tpTMS site was involved in statistically significant interaction effects (GL*tpTMS site and GL*tpTMS site*tpTMS timing, see Table 2A), explained by rather small, yet borderline significant, differences between the effects of tpTMS of MT/V5, V6 and IFG compared to the Vertex, which, did not survive the restrictive Bonferroni correction we adopted for the post-hoc tests, but showed p-values lower than the 0.05 cut-off with the less restrictive LSD correction (see the inset colored graphs associated to Figure 6A). These effects consisted of the underestimation of the landing location of 0g trajectories following tpTMS600 of MT/V5 [*t*_(240)_ = −2.07, CI = (1.05 −7.34), *p* = 0.040, LSD adjusted] and V6 [*t*_(240)_ = −2.17, CI = [0.84 −6.57), *p* = 0.031, LSD adjusted], and overestimation of 1g trajectories following tpTMS600 of IFG [*t*_(240)_ = 2.38, CI = (−0.51 6.19), *p* = 0.018, LSD adjusted]. Finally, as observed with the temporal accuracy, the statistically significant two-way interaction effect GL*tpTMS timing indicated that *tpTMS600* produced larger differences than *tpTMS100* in the spatial accuracy of 0g compared to 1g responses. Instead, the precision of the mouse placement was not significantly affected by tpTMS (Figure 6B and Table 2A).

### Occluded session

**FIGURE 7:**
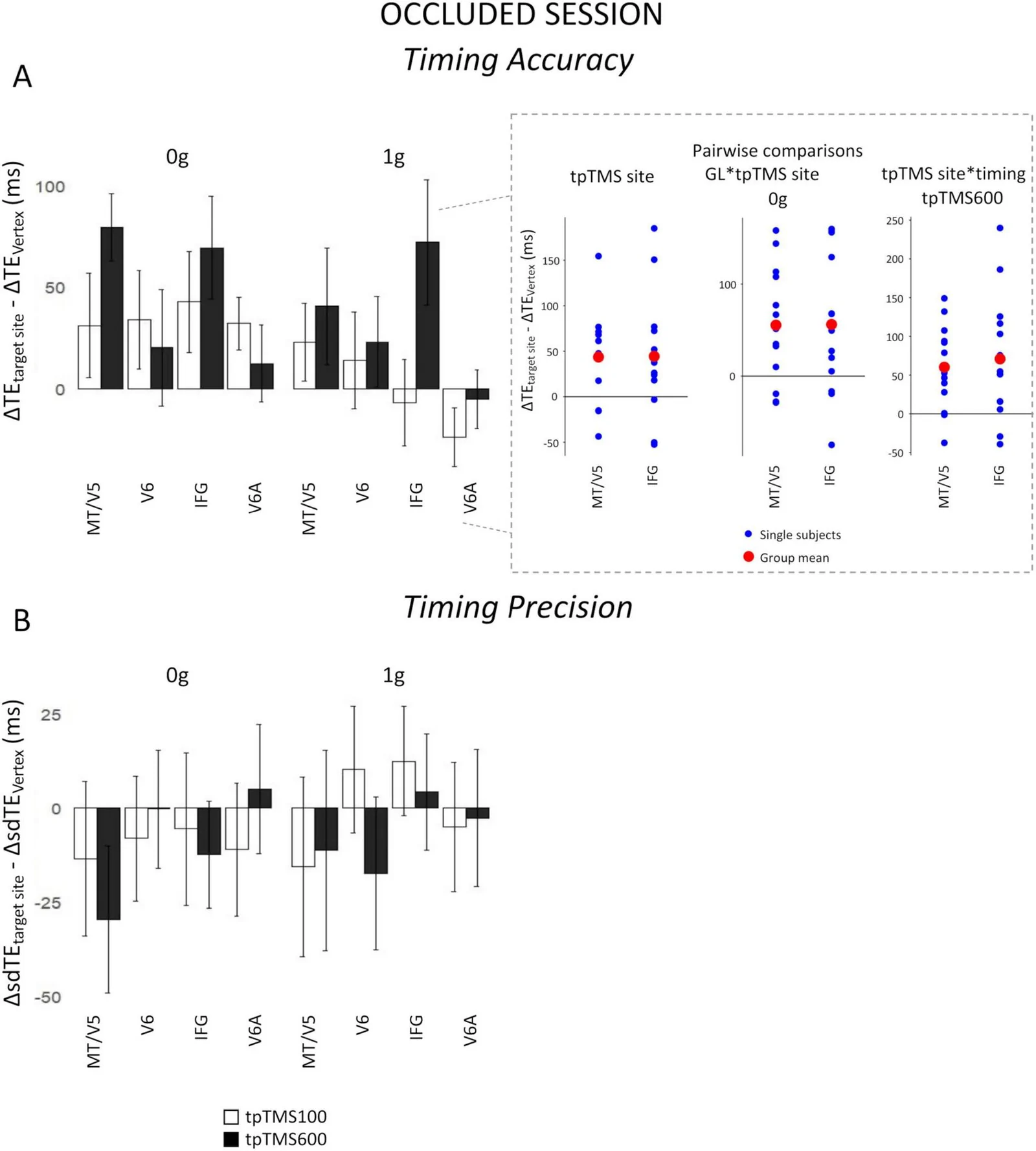
Effects of tpTMS of the four target cortical areas on the interceptive timing observed during the *Occluded Session.* Same layout as Figure 5.

Table 2B reports the results of the GLMM analysis carried on the *Occluded Session* datasets. We found that, with occluded trajectories, the interceptive timing accuracy was strongly influenced by tpTMS timing, with *tpTMS100* and t*pTMS600* producing, respectively, earlier and later responses relative to the no-stimulation baseline. tpTMS site also emerged as a strong predictor. Thus, we applied planned post-hoc pairwise comparisons to evaluate whether the main effect of tpTMS site was accounted for by differences between the effects of tpTMS of the target areas compared to the Vertex, considering that Vertex stimulation, on average, produced slightly earlier responses (mean ΔTE = −24.40 ± 14.01 SEM). The results of these *post-hoc* tests, illustrated by the colored inset graphs of Figure 7A, indicated genuine effects of tpTMS for MT/V5 and IFG [*t*_(240)_ = 3.24, CI = (5.43 81.79), *p* = 0.006 and *t*_(240)_ = 2.46, CI = (−5.39 94.22), *p* = 0.029, respectively], which delayed greatly the interceptive timing compared to the Vertex. The statistically significant two-way interactions tpTMS site*tpTMS timing and GL*tpTMS site indicated further that site-specific effects depended on whether tpTMS was applied after the trajectory perturbation or occlusion and on the trajectory congruence with gravity. Notably, pairwise comparisons between target sites and the Vertex applied to these interaction effects showed that stimulation of MT/V5 and IFG delayed significantly the interceptive responses compared to the Vertex with 0g trajectories [MT/V5: *t*_(240)_ = 3.29, CI = (7.80 103.03), *p* = 0.004; IFG: *t*_(240)_ = 2.77, CI = (−0.59 112.94), *p* = 0.018] and with *tpTMS600* [MT/V5: *t*_(240)_ = 4.10, CI = (18.61 101.72), *p* < 0.001; IFG: *t*_(240)_ = 3.24, CI = (10.45 131.32), *p* = 0.003; see the rightmost inset graphs associated to Figure 7A]. Instead, the interceptive timing precision was affected significantly only by tpTMS timing, with greater response timing variability following *tpTMS100* compared to *tpTMS600* (Figure 7B).

**FIGURE 8:**
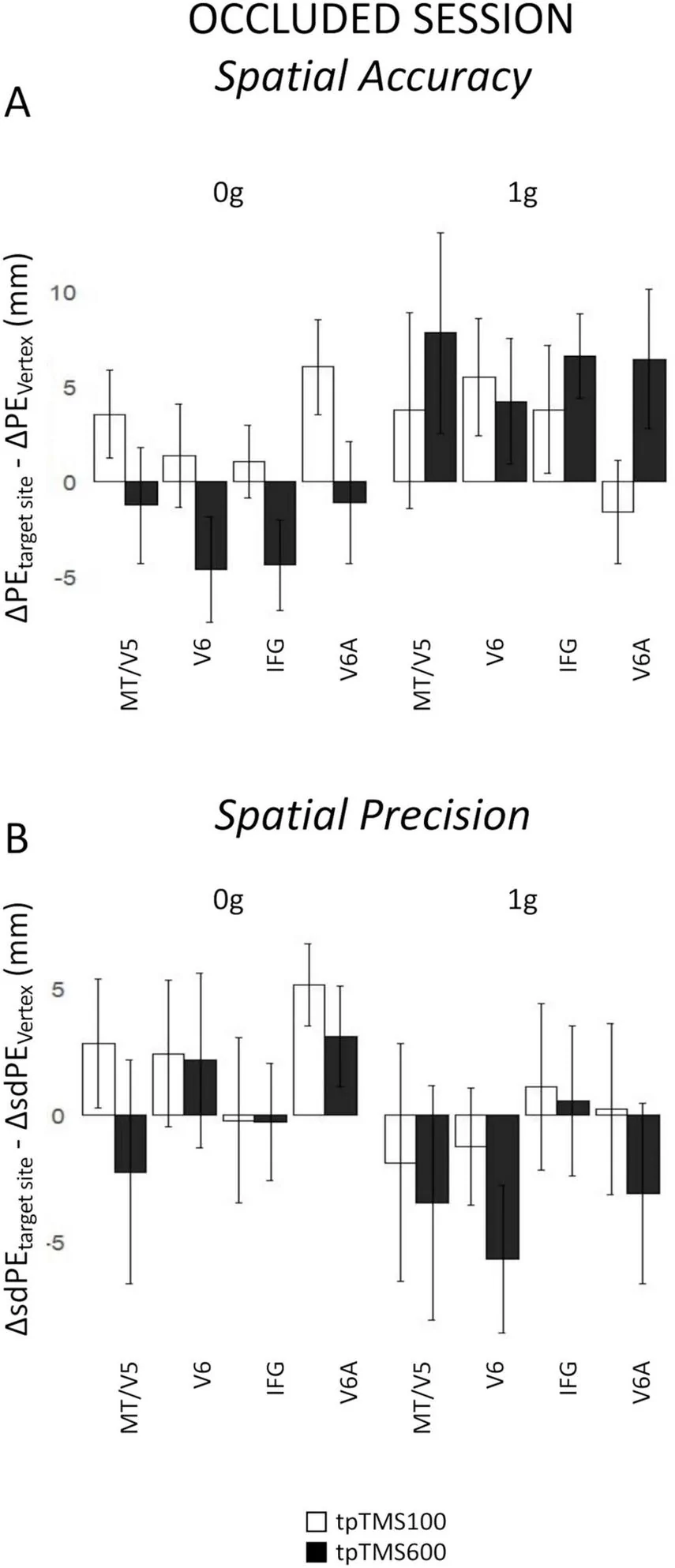
Effects of tpTMS of the four target cortical areas on the interceptive positioning observed during the *Occluded Session.* Same layout as Figure 6.

The spatial accuracy of the interceptive responses depended primarily on tpTMS timing, with stronger effects of *tpTMS100* relative to *tpTMS600*. The stronger effects of *tpTMS100* were particularly evident with perturbed 0g compared to unperturbed 1g trajectories, accounting for the significant two-way interaction GL*tpTMS timing (see Figure 8A and Table 2B). We also found a statistically significant two-way interaction GL*tpTMS site. However, *post-hoc* pairwise comparisons between TMS sites applied to this interaction indicated stronger changes in the spatial accuracy following Vertex than target areas’ stimulation, implying nonspecific effects (Figure 8A). Similarly, the spatial precision of the mouse cursor placement (ΔsdPE) was significantly influenced by the two-way interaction tpTMS site*tpTMS timing, but post-hoc tests did not reveal significant differences between the target areas and the Vertex, suggesting that also this effect was, likely, nonspecific (Figure 8B).

## Discussion

We investigated the contribution of four cortical areas to the control of manual interceptive actions: visual motion areas MT/V5 and V6, the visuomotor area V6A, and a premotor cortical site in the inferior frontal gyrus (IFG), belonging to the vestibular network. Specifically, we assessed the causal involvement of these regions both when visual information was fully available and when extrapolation mechanisms were required due to the temporary target occlusion. Our findings revealed distinct and complementary roles of these cortical regions in the temporal control of interception, as well as in visual extrapolation processes.

### Contributions of visual areas MT/V5 and V6

A primary objective of the study was comparing the contributions of visual areas MT/V5 and V6. We found that their stimulation produced rather similar effects on the interceptive responses when targets were fully visible, as expected based on previous neuroimaging work (Pitzalis et al., 2010, 2013a,c), but an evident functional dissociation emerged with visual occluded targets.

Indeed, stimulation of area MT/V5 produced the most robust and consistent effects, by altering primarily the interception timing in response to both visible and occluded trajectories, consistently with the well-established role of this cortical area in the processing of visual motion and of the timing of visual events (Bosco G. et al., 2008; Delle Monache et al., 2017; Battelli et al., 2007, 2008; Bueti et al., 2010; Salvioni et al., 2013, Harvey et al., 2020; Tonoyan et al., 2022; Centanino et al., 2024). Notably, during the *Occluded Session*, tpTMS applied immediately after the target disappearance delayed the interceptive responses to both 1g and 0g trajectories (with 0g responses affected to a greater extent), implying an involvement of MT/V5 in motion extrapolation. This result appears in line with neuroimaging evidence that fMRI signals in this area may remain elevated when observing moving targets even during periods of visual occlusion (Olson et al., 2004).

Moreover, the opposite effects on the interceptive timing observed with visible and occluded motion may suggest context-dependent contributions of area MT/V5, which may be in line with evidence of context-dependent integration of sensory and predictive information in interceptive and oculomotor control (Márquez and Treviño, 2025). The earlier interceptive responses observed following MT/V5 disruption when visual information of the target motion is continuously available has been, in fact, interpreted as evidence that MT/V5 activity can modulate central processing delays (Bosco G. et al., 2008; Nijhawan and Wu, 2009; Nijhawan, 2002; Nijhawan and Kirschfeld, 2003; Maus et al., 2013a; Hogendoorn, 2020). Consistent with this interpretation, here we found that also tpTMS of a visual area downstream of MT/V5, like area V6, produced similar shortening of the interceptive timing during the *Visible Session*.

With respect to the delayed responses when tpTMS was applied to MT/V5 in the absence of visual signals, we may hypothesize that MT/V5 could hold a working memory buffer of visual motion information processed before the target disappearance, which is, then, relayed to intraparietal areas (e.g., LIP) involved more critically in visual motion extrapolation processes (Salvioni et al., 2013; Bisley et al., 2001, 2004; Shuwairi et al., 2007). Another interpretation, which does not necessarily exclude the previous one, is that MT/V5 stimulation could have also influenced trans-synaptically the intraparietal areas involved in the visual extrapolation processes (Ferreri et al., 2011; Garcia et al., 2011; Fox et al., 2012; Giambattistelli et al., 2014; Hallett et al., 2017; Bossus et al., 2026). Finally, these two hypothetical mechanisms are also compatible with the possibility of recurrent mechanisms between area MT/V5 and intraparietal areas (Siegel et al., 2015).

Unlike the stimulation of MT/V5, which produced effects with both fully visible and occluded trajectories, disrupting V6 activity altered the interceptive responses only when trajectories were visible, suggesting that this area may not be involved in the visual extrapolation of occluded motion. The effects on the interceptive timing concerned only the accuracy, with significant effects emerging only with *tpTMS600*. This result may be compatible with the idea that V6 represents a processing stage downstream of MT/V5 (Galletti et al., 2001; Tosoni et al., 2015), given that effects of MT/V5 stimulation during the *Visible Session* were observed only with *tpTMS100*.

### Dissociation between V6 and V6A contributions

While tpTMS over V6 altered significantly the interceptive performance, stimulation of V6A did not seem to produce statistically significant effects compared to the Vertex. Indeed, a direct comparison between the effects of stimulation of V6 and V6A during the *Visible Session*, when significant effects of area V6 compared to the Vertex were evident in the GLMM analysis of the interceptive accuracy, indicated significantly greater effects for V6 than V6A [*t*_(240)_ = 2.56, CI = (2.37 19.67), *p* = 0.014]. This result was somewhat unexpected, given that TMS applied to V6A produced causal effects on goal directed arm movements with reduced movement accuracy toward targets located further away from the plane of gaze fixation, impairment of online reaching corrections and altered grip aperture during grasping (Breveglieri et al., 2021, 2024). Moreover, increased functional connectivity within the superior parietal lobule, including area V6A, has been reported during a task requiring temporal and spatial predictions of occluded parabolic trajectories (Zbären et al., 2024).

On one hand, the negative result emerging from our experiments, in part, may reflect specific task constraints inherent to the experimental design. First, we presented 2D ball trajectories confined to the frontal plane, whereas earlier TMS studies on grasping movements found stronger effects of V6A stimulation for targets displaced in depth (Breveglieri et al., 2021). Second, the interception task did not require physical interaction with the ball, while it is possible that V6A activity may be primarily engaged in preparation to the physical interaction with objects, such as in grasping movements. In other words, using 2D trajectories and removing the physical contact with the objects may have downplayed V6A’s role. Adoption of more immersive setups involving physical interaction with the moving objects may help clarifying this issue. On the other hand, the lack of effects of V6A stimulation may be in line with the evidence that V6 and V6A, although contiguous, belong to separate cytoarchitectonic areas, BA19 and BA7, respectively, thereby reinforcing their functional distinction (Gamberini et al., 2020). Moreover, it may represent strong indirect evidence that the effects observed with magnetic stimulation of the other target sites (i.e., V6, MT/V5, and IFG) were related to genuine disruptions of their activities.

### Contribution of IFG

The IFG site was selected based on its fMRI activation by gravity-congruent visual motion (Indovina et al., 2005). This premotor region can be considered part of the vestibular network, a distributed cortical and subcortical system integrating vestibular signals, which has been also associated with the elaboration and storage of the internal model of gravity (Brandt and Dieterich, 1999; Lopez et al., 2012; Lobel et al., 1998; Indovina et al., 2020; Raiser et al., 2020; Delle Monache et al., 2021). Indeed, previous TMS studies targeting a core vestibular region in the TPJ, have shown to affect selectively the interception timing in response to gravity-congruent motion (Bosco G. et al., 2008; Delle Monache et al., 2017). However, there are no studies, to our knowledge, that have investigated the role of this specific premotor cortical site in the control of manual interception actions.

Overall, the present results support this possibility. IFG stimulation produced consistent effects only with occluded motion, by delaying the interceptive responses to both 1 g and 0g trajectories (although those to 0g motion to a greater extent), much alike the effects of MT/V5 stimulation. This finding is consistent with the results of a previous neuroimaging study, reporting significant bilateral activation in the inferior frontal gyrus at similar coordinates as the present site by contrasting the fMRI responses to occluded compared to unoccluded motion (Shuwairi et al., 2007). This may imply a role for IFG in maintaining a working memory representation of the occluded target, which may be instrumental in driving the activity of downstream cortical motor neurons for timing the motor response (Merchant et al., 2004a,b; Marinovic et al., 2011; Liu and Pleskac, 2011; Curtis and D’Esposito, 2006).

### Target areas’ contributions on the spatial aspects of the interceptive response

Stimulation of the target areas did not produce significant differences on the mouse cursor positioning compared to the Vertex stimulation according to the restrictive statistical cut-offs we adopted. In effect, also in a previous study using the same experimental set-up, we found that tpTMS effects on mouse positioning were much less consistent compared to those observed for the response timing (Delle Monache et al., 2017). A possible explanation for the less consistent effects of tpTMS on the mouse positioning may concern the motor strategy generally adopted by subjects, who displaced the mouse cursor close to the presumed interception location early on during the ball trajectory—particularly during the occluded session—and performed only small positional adjustments afterwards (Bosco et al., 2012). Nevertheless, we noted that the statistically significant interactions involving the predictor tpTMS site during the *Visible Session* were accounted by small, yet systematic, differences between the effects of MT/V5, V6 and IFG stimulation compared to the Vertex, which satisfied the p < 0.05 cut-off by applying the less restrictive LSD correction. These effects consisted of underestimation of 0g trajectories following stimulation of visual areas MT/V5 and V6 and overestimation of 1g trajectories following IFG stimulations. These observations may be suggestive in two respects, albeit pending further support from additional studies: (1) IFG selectivity for 1g trajectories appears compatible with a role in the processing of internalized information about gravity, in line with its affiliation to the vestibular network (Indovina et al., 2005, 2020; Delle Monache et al., 2021); (2) the spatial underestimation of 0g trajectories following MT/V5 and V6 stimulation may be consistent with increased reliance on predictive signals when visual motion information is disrupted (Márquez and Treviño, 2024). Specifically, it may suggest greater reliance on internalized gravity information (which, for constant velocity trajectories, would predict a landing location closer to the center of the visual scene), in line with the antagonistic “push–pull” relationship between visual information and gravity a priori suggested by De Sá Teixeira et al. (2019) to account for a similar observation during perceptual localization of vanishing moving targets.

### Distributed cortical processing for the control of interceptive actions

**FIGURE 9:**
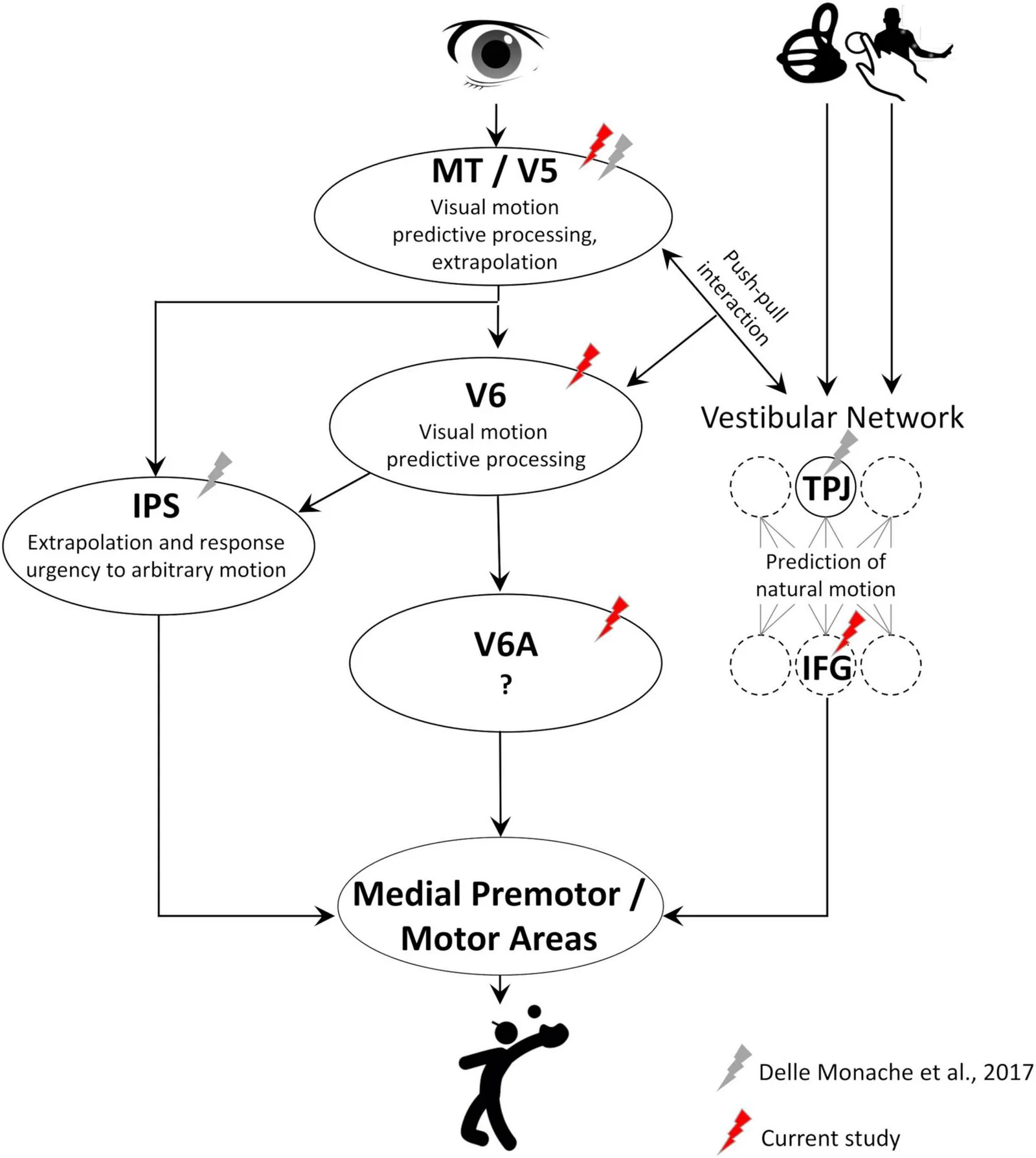
Schematic representation of the hypothetical organization within the cortical network underlying manual interception of occluded motion originally proposed by Delle Monache et al. (2017) and updated with the evidence emerging from the present study. Red and grey thunderbolts indicate the cortical areas targeted by tpTMS in the present (MT/V5, V6, V6A, IFG) and in the earlier study (MT/V5, TPJ, IPS), respectively. The arrows connecting elements of the cortical network do not represent direct anatomical connections but indicate simply the hypothetical flow of information. The (?) symbol associated to area V6A refers to the possibility, not tested by the present study, that V6A may contribute to the interceptive control in the presence of conditions, such as 3D visual motion and direct physical interaction with the object.

The present findings may provide additional elements to the parallel distributed organization scheme proposed by Delle Monache et al. (2017) for the network of cortical areas potentially involved in the interception of visually occluded projectile motion. Figure 9 updates the original scheme from Delle Monache et al. (2017) with the main findings emerging from the present study. According to this hypothetical scheme, area MT/V5 would represent an early processing stage of visual motion information responsible for feeding predictive timing information to downstream areas, such as V6, posterior parietal cortical areas, including those in the intraparietal sulcus (IPS), involved in visuomotor transformations, and vestibular network areas in the TPJ (Bosco G. et al., 2008; Battelli et al., 2007; Bueti et al., 2008, 2010; Heed et al., 2011; Salvioni et al., 2013; Delle Monache et al., 2017; Harvey et al., 2020; Tonoyan et al., 2022; Centanino et al., 2024; Márquez and Treviño, 2026). Our current results may also suggest that, when the object motion becomes temporarily occluded, area MT/V5 may supply a memory signal of the object motion prior to its disappearance to downstream areas directly involved in the extrapolation processes, such as IPS (Assad and Maunsell, 1995; O’Reilly et al., 2008; Shuwairi et al., 2007). Areas V6, the other visual motion area investigated here, would contribute to the predictive processing of the interceptive responses only in the presence of visual information, backing up, to some degree, the role of area MT/V5.

Regarding the extrapolation of the occluded ball motion, the earlier scheme suggested a parallel organization with respect to the causal nature of the target motion, based on the finding that disruption of IPS activity altered significantly the responses to 0g and 2g motion without affecting those to motion congruent with gravity, whereas stimulation of TPJ affected only responses to gravity-congruent motion. This led to the proposal that IPS would be critical for the extrapolation of arbitrary visual motion, whereas representation of occluded 1g motion may occur in vestibular network areas other than TPJ, because its stimulation after the target disappearance did not alter the interceptive performance. Here we showed that the premotor vestibular site IFG did affect causally the interceptive timing when its activity was disrupted right after the ball disappearance, however showing a slight selectivity for 1g motion only with respect to the spatial aspects of the interceptive response. In this general scheme, IFG may combine predictive information about the occluded object motion from parietal areas, such as IPS, with gravity a priori information processed in the vestibular network, driving motor cortical activity to produce timed and spatially accurate responses. Finally, although our current study did not reveal causal effects of the stimulation of visuomotor area V6A, we cannot rule out that it may be more engaged with tasks involving visual motion in depth and physical interactions with the interceptive object, which may be object of future studies.

### Limitations of the study

One potential limitation of the study concerned the impact of motor facilitation effects of auditory and somatosensory artifacts of the tpTMS delivered during the interceptive task (Pascual-Leone et al., 1992; Terao et al., 1997). To minimize the impact of these effects we adopted two countermeasures: first, we mitigated the auditory artifacts of the coil discharge by covering them with white noise; second, we used the Vertex as control stimulation site and related the difference in interceptive behavior observed following the stimulation of the target areas with those observed following Vertex stimulation. Although Vertex stimulation is a viable control condition, there is, however, evidence that the intensity of the perceived somatosensory and auditory artifacts can vary depending on the location of the TMS site on the scalp, being highest in frontal regions (Meteyard and Holmes, 2018). Thus, in our experiments, somatosensory and residual auditory artifacts, might have influenced more the effects of IFG stimulation than those of the other TMS sites, including the Vertex. At least three observations can rule out this possibility, indicating genuine effects of cortical disruptions: (1) The distribution of tpTMS effects across scalp sites was not consistent with Meteyard and Holmes’s (2018) mapping of the artifactual effects, because the most consistent effects were observed, during both experimental sessions, with area MT/V5, while IFG affected significantly the interceptive timing only during the *Occluded Session*; (2) the significant effects of tpTMS of IFG during *Occluded Session* consisted of delayed interceptive responses, whereas the effects reported for TMS auditory and somatosensory artifacts during the execution of timed motor tasks, including those of Vertex stimulation during manual interception, confirmed also by the current study, generally, concern motor facilitation with earlier response timing (see for example, Pascual-Leone et al., 1992; Terao et al., 1997; Delle Monache et al., 2017); (3) tpTMS of V6 and V6A, two very close scalp sites for which the strength of TMS artifacts should be comparable, produced effects of significantly different amplitude on the interceptive responses.

## Conclusion

In summary, our findings provided causal evidence for context specific functional differences among four cortical regions in the control of manual interceptions, which seem compatible with distributed information processing: MT/V5 may provide context-dependent contributions, by affecting central processing delays if visual information is continuously available, while contributing to the visual extrapolation processes if targets are transiently occluded; V6 may play a role only with continuously available visual information in modulating the central processing delays downstream of area MT/V5; IFG might integrate predictive signals based on visual information and on internalized gravity information. Together, these results may refine current models of the cortical control of manual interception.

## Data Availability

The raw data supporting the conclusions of this article will be made available by the authors, without undue reservation.
